# Comparative evaluation of IV ketorolac and IV paracetamol in laparoscopic surgeries: a systematic review and meta-analysis

**DOI:** 10.1097/MS9.0000000000005740

**Published:** 2026-10-14

**Authors:** Muhammad Asad Jahangir, Usama Ahmad, Mehrab Naeem, Maaz Amir, Ayesha Wasim, Muhammad Huzaifa Khalil, Muhammad Salayh Khan, Muhammad Azaan Khan, Muhammad Sarmad, Muhammad Azfar, Abdul Haseeb Hasan, Ahmad Hassan

**Affiliations:** a Department of Medicine, King Edward Medical University, Department of Medicine, Mayo Hospital, Lahore, Punjab, Pakistan; b Department of Medicine, Oli Health Magazine Organization, Research and Education, Kigali, Rwanda

**Keywords:** IV Ketorolac, IV Paracetamol, laparoscopic Surgeries, Meta-analysis

## Abstract

**Background:**

Effective postoperative pain control is essential for enhanced recovery after laparoscopic surgery. Intravenous ketorolac and paracetamol are widely used non-opioid agents in multimodal analgesia, but their relative efficacy and safety remain controversial.

**Methods:**

Following Preferred Reporting Items for Systematic Reviews and Meta-Analyses 2020 guidelines, databases were searched through September 2025 for randomized and comparative studies of intravenous (IV) ketorolac versus IV paracetamol. Pooled effect estimates with 95% confidence intervals were calculated using RevMan 5.4. The primary outcome was postoperative visual analog scale (VAS) pain scores; secondary outcomes included the timing and use of rescue analgesia, hemodynamic parameters, and adverse events.

**Results:**

Eight studies encompassing 540 patients met inclusion criteria. IV ketorolac significantly reduced VAS scores at 2 hours (MD = −0.95; 95% CI: −1.17 to −0.74; *P* < 0.00001), 4 hours (MD = −0.99; 95% CI: −1.62 to −0.35; *P* = 0.002), and 8 hours (MD = −0.45; 95% CI: −0.78 to −0.13; *P* = 0.006) postoperatively compared with IV paracetamol. The time to first rescue analgesia was significantly longer with ketorolac (MD = 1.93; 95% CI: 1.26–2.61; *P* < 0.00001). Postoperative vomiting occurred less frequently with ketorolac, while other adverse events and hemodynamic parameters were comparable between the groups. Substantial statistical heterogeneity was present for some outcomes.

**Conclusion:**

Intravenous ketorolac provides better early postoperative analgesia and longer-lasting pain relief than intravenous paracetamol following laparoscopic surgery, without increased adverse events. Vomiting was also less frequent with ketorolac. Both agents showed acceptable short-term safety and may be used for multimodal, opioid-sparing analgesia guided by patient and procedural factors.

## Introduction

Postoperative pain is common, and effective management is essential to prevent the negative consequences of acute postsurgical pain and to support a smooth return to normal function. Inadequate management of postoperative pain might raise the risk of cardiovascular and pulmonary issues while delaying recovery and discharge. Opioids have long been the mainstay of postoperative pain relief, but concerns about overuse and adverse reactions have led to a trend toward multimodal approaches that combine non-opioid medications with regional anesthetic techniques^[^1^]^. Because it allows for smaller incisions, quicker recovery, a lower risk of infection, and better results, laparoscopic surgery (LS) has revolutionized surgical practice globally. Since intravenous (IV) paracetamol and ketorolac are commonly used in Enhanced Recovery After Surgery (ERAS) protocols, effective pain management remains crucial, making the relative efficacy of these medications an important clinical consideration^[^2,3^]^.

HIGHLIGHTS

- This systematic review and meta-analysis compared intravenous (IV) ketorolac and IV paracetamol for postoperative analgesia in laparoscopic surgeries.
- Eight studies (*n* = 540 patients) from India, Venezuela, Bangladesh, and Nepal were included.
- IV ketorolac significantly reduced visual analog scale pain scores at 2, 4, and 8 hours postoperatively compared with IV paracetamol.
- Time to first-rescue analgesia was significantly longer with IV ketorolac.
- No significant differences were observed in the total rescue analgesic consumption or in the number of patients requiring rescue analgesia.
- Hemodynamic parameters (mean arterial pressure and heart rate) were comparable between the two groups.
- The incidence of postoperative vomiting was significantly lower with IV ketorolac; nausea rates were similar.
- Sensitivity analysis confirmed the robustness of ketorolac's early postoperative analgesic superiority.
- Both agents are safe and suitable for opioid-sparing multimodal analgesia in laparoscopic surgery.
- Analgesic selection should be individualized based on patient comorbidities and surgical context.

IV paracetamol (acetaminophen) is a widely used analgesic for short-term relief of mild to moderate pain, and it can also be paired with opioids for managing more severe pain. In LS, it has shown particular benefit by easing postoperative discomfort and reducing the need for opioid medications. It is believed to act through central cyclo-oxygenase inhibition and modulation of serotonergic pathways, resulting in a rapid onset and a reassuring safety profile^[^4^]^.

Ketorolac tromethamine (Toradol), a nonsteroidal anti-inflammatory drug (NSAID), is also extensively used in postoperative care and provides analgesia through peripheral prostaglandin inhibition. Laparoscopic cholecystectomy studies have shown that intraoperative or preoperative IV ketorolac decreases pain scores and lessens the requirement for rescue analgesia. Ketorolac, in contrast to paracetamol, has known hazards, including bleeding, renal impairment, and gastrointestinal complications, which call for careful patient selection and dosing^[^5,6^]^.

Comparative effectiveness of IV paracetamol and ketorolac in the context of LS remains clinically important. The few head-to-head trials available vary in design, differing in procedure type, timing of administration, and use of additional analgesics^[^7^]^. Some report comparable pain relief between the two drugs, while others suggest one may have an edge, particularly when used as part of a multimodal approach such as premedication in laparoscopic cholecystectomy^[^8^]^. Beyond individual trials, narrative reviews and quantitative syntheses affirm that IV paracetamol’s opioid-sparing effect is credible but variable by surgery and timing; ketorolac’s benefits are procedure-dependent and must be balanced against risks (e.g., bleeding, renal effects)^[^9^]^.

Despite the fact that narrative reviews and individual studies offer valuable information, no systematic review or meta-analysis has precisely compiled the data comparing IV paracetamol with IV ketorolac in laparoscopic procedures^[^10^]^. Since both medications are already commonly used in practice and pain management is essential to ERAS protocols, it is evident that their relative efficacy and safety need to be clarified. By combining data from multiple evidence-based studies and examining pain intensity [visual analog scale (VAS)] as the primary endpoint, as well as the timing and need for rescue analgesics, hemodynamic parameters, and possible side effects, this meta-analysis aims to close that gap. The knowledge acquired will facilitate the selection of safe, efficient, and opioid-sparing regimens that promote recovery and improve the overall experience for patients undergoing LS, enabling anesthesiologists and surgeons to make better decisions.

## Materials and methods

The study was conducted in accordance with the Preferred Reporting Items for Systematic Reviews and Meta-Analyses (PRISMA) guidelines^[^11^]^.

### Search strategy

A thorough literature search was conducted across three principal databases (PubMed, Google Scholar, and the Cochrane Library) to find all pertinent research.

Publications were obtained from the database’s inception through September 2025. The search method used both Medical Subject Headings and free-text keywords, including terms such as “intravenous ketorolac,” “intravenous paracetamol,” “laparoscopic surgeries,” “postoperative analgesia.” Using the Boolean operators “AND” and “OR” the search was modified to balance sensitivity and specificity. Detailed, database-specific search strategies are provided in the Supplementary file.

### Eligibility criteria

Studies were included based on the following criteria: original studies (randomized controlled trials (RCTs), cohort studies, case-control studies, cross-sectional studies); adult patients undergoing LS; studies with mixed surgical populations were included if separate data for laparoscopic procedures were available or extractable; administration of IV ketorolac or IV paracetamol preoperatively or perioperatively; comparison between IV ketorolac and IV paracetamol; trials with additional comparator arms (e.g., placebo, other analgesics) were included only if a head-to-head comparison between IV ketorolac and IV paracetamol was reported; studies in a non-English language with an English translation available. Exclusion criteria were: case reports, reviews, editorials, and conference abstracts without full data; patients younger than 18 years; procedures other than LS; use of oral, intramuscular, rectal, or other non-IV formulations of ketorolac or paracetamol, and studies published in a non-English language with no translation available or with full texts unavailable.

### Data extraction

Using a standard Google Sheets template, two independent reviewers retrieved study-level data and recorded important factors such as the author’s name, research design, publication year, study region, overall sample size, sample sizes for each group, and baseline characteristics including mean age, mean weight, males and females, heart rate (HR), and mean arterial pressure (MAP). The primary outcome was the VAS score, and the secondary outcomes included time to first rescue analgesia, total consumption of rescue analgesics, the number of patients requiring rescue analgesia, MAP and HR 60 minutes after intubation, HR 2 hours postoperatively, and the incidence of adverse effects such as nausea and vomiting. Discrepancies were resolved through discussion or adjudication by a third reviewer.

### Quality assessment

The risk of bias in the included RCTs was evaluated using the Cochrane Collaboration’s Risk of Bias 2.0 (RoB 2.0) tool^[^12,13^]^. This structured assessment encompasses five key domains: (1) bias arising from the randomization process, including adequacy of sequence generation and allocation concealment; (2) bias due to deviations from intended interventions, reflecting adherence to assigned treatments and the effectiveness of blinding; (3) bias due to missing outcome data, considering the extent, reasons, and handling of incomplete data; (4) bias in outcome measurement, including blinding of outcome assessors and objectivity of outcome measures; and (5) bias in the selection of reported results, assessing the consistency between prespecified outcomes and those reported. For non-randomized studies, the Newcastle–Ottawa Scale^[^14^]^ was utilized to assess methodological quality across three domains: selection of participants, comparability of cohorts, and ascertainment of outcomes. To ensure methodological rigor, two independent reviewers conducted the assessments in accordance with the Cochrane Handbook guidelines, with discrepancies resolved through discussion or consultation with a third reviewer. In addition to domain-level judgments (“low risk,” “some concerns,” or “high risk”), an overall risk-of-bias judgment was assigned for each study. Furthermore, study design characteristics, participant selection processes, and inclusion and exclusion criteria were carefully reviewed to assess external validity and potential selection bias. Particular attention was given to randomization methods, baseline comparability of study groups, and the use of blinding to minimize performance and detection bias.

### Statistical analysis

All statistical analyses were performed using Review Manager (RevMan) version 5.4. For each outcome, pooled estimates were calculated as odds ratios (ORs) for categorical variables and mean differences (MDs) with standard deviations (SDs) for continuous variables, accompanied by 95% confidence intervals (CIs). In every analysis, IV ketorolac was entered as the experimental arm and IV paracetamol as the comparator, and all effect estimates were computed as ketorolac minus paracetamol. Accordingly, for pain intensity, hemodynamic, and rescue-analgesic-consumption outcomes, a negative MD favors IV ketorolac, whereas for time to first rescue analgesia, a positive MD favors IV ketorolac; for dichotomous outcomes, an OR below 1.0 favors IV ketorolac. This orientation applies to every forest plot presented in the main text and in the supplementary appendices. The *I*^2^ statistic was used to evaluate the studies’ heterogeneity; low heterogeneity was indicated by *I*^2^ values <50%, moderate heterogeneity by *I*^2^ values 50–75%, and considerable heterogeneity by *I*^2^ values >75%. For heterogeneity greater than 50%, a random-effects model was used, whereas for heterogeneity less than 50%, a fixed-effect model was used. To evaluate the stability of the results, sensitivity analyses were conducted. Each study was sequentially excluded to determine whether its exclusion led to a reduction in heterogeneity. Studies whose removal resulted in decreased heterogeneity were highlighted in a table, along with the extent of heterogeneity reduction. To explore potential sources of clinical heterogeneity, two pre-specified subgroup analyses were performed: one stratifying studies by study design (RCTs and cohort studies) and one stratifying studies by type of LS (laparoscopic cholecystectomy, gynecologic LS, and any LS). The stratification by study design was purposefully utilized as a sensitivity analysis to present the randomized evidence independently. This ensured that the inclusion of non-randomized data, which were incorporated to capture the totality of available comparative evidence, did not skew the primary RCT findings. Statistical significance was defined as a P-value below 0.05. Egger’s test, a regression-based statistical method, is usually used to formally assess the presence of publication bias. However, in this study, Egger’s regression test was not applied, consistent with methodological consensus that it is unsuitable for meta-analyses with fewer than ten included studies. Small-study effects and publication bias, therefore, could not be formally excluded, and this is acknowledged as a limitation.

## Results

### Selected studies and study characteristics

Out of 1843 studies initially identified from various databases, one non-randomized comparative study (described by its authors as cross-sectional) and seven RCTs involving a total of 540 participants were ultimately included. Study selection followed the PRISMA 2020 guidelines, as depicted in Figure 1. Key study characteristics are outlined in Table 1, while baseline characteristics are highlighted in Table 2. Across all included studies, participants were divided into two groups: those who received IV ketorolac (*n* = 270, 50%) and those who received IV paracetamol (*n* = 270, 50%). Studies were conducted in four countries: Venezuela, India, Bangladesh and Nepal. In the study-design subgroup analyses, the non-randomized study is labeled the “cohort study” subgroup, consistent with the Newcastle–Ottawa Scale used for its appraisal.

**Figure 1.**
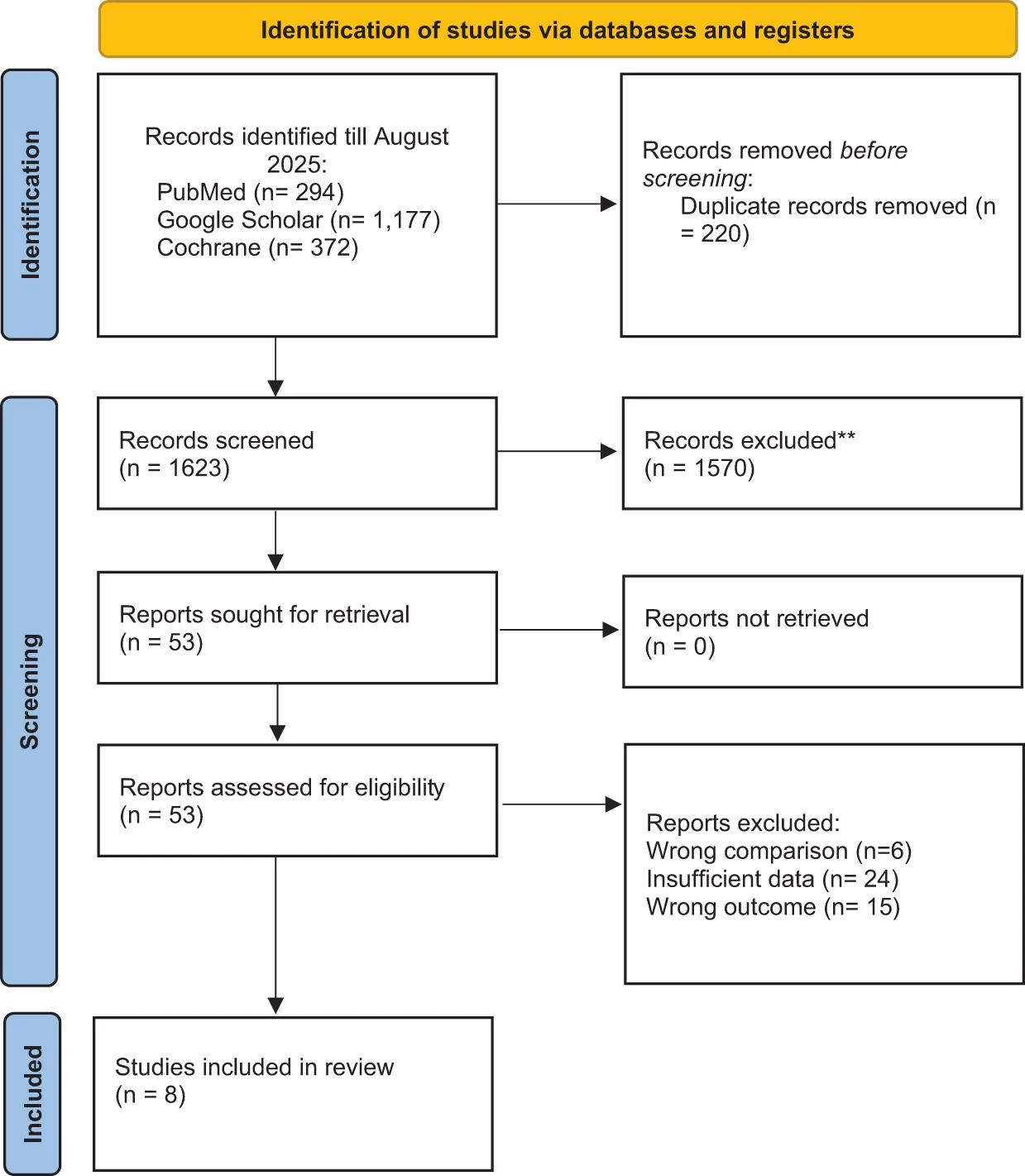
PRISMA flow diagram.

**Table 1:** Study characteristics.

Study author	Year	Country	Study design	Population iv ketorolac (*n*)	Population iv paracetamol (*n*)
Rastogi *et al*^[^15^]^	2016	India	Randomized controlled trial	45	45
Khadri *et al*^[^16^]^	2024	India	Randomized controlled trial	24	24
Medina-Vera *et al*^[^17^]^	2016	Venezuela	Randomized controlled trial	49 (50)^a^	49 (50)^a^
Nisarga *et al*^[^18^]^	2024	India	Randomized controlled trial	27	27
Rajoria *et al*^[^19^]^	2023	India	Randomized controlled trial	30	30
Shaheen *et al*^[^20^]^	2017	Bangladesh	Randomized controlled trial	30	30
Neupane *et al*^[^21^]^	2024	Nepal	Cross-sectional study	35	35
Ramakrishnan *et al*^[^22^]^	2017	India	Randomized controlled trial	30	30

IV, intravenous.

^a^ One patient lost to follow-up.

**Table 2:** Baseline characteristics.

	Age	Sex		Mean weight	Heart rate	MAP
Study	Mean SD (years)	Male (*n*)	Female (*n*)	Mean SD (kg)	Mean SD (bpm)	Mean SD (mm of Hg)
Rastogi *et al*^[^15^]^						
IV ketorolac	39.9 ± 16.22	12	18	56.40 ± 2.6	84.57 ± 7.12	85.50 ± 6.51
IV paracetamol	41.43 ± 15.73	14	16	56.50 ± 2.5	81.93 ± 6.34	87.83 ± 5.35
Khadri *et al*^[^16^]^						
IV ketorolac	36.08 ± 11.28	NA	NA	NA	NA	NA
IV paracetamol	40.54 ± 12.22	NA	NA	NA	NA	NA
Medina-Vera *et al*^[^17^]^						
IV ketorolac	38 ± 11	38	12	NA	NA	NA
IV paracetamol	41 ± 10	36	14	NA	NA	NA
Nisarga *et al*^[^18^]^						
IV ketorolac	37.52 ± 10.79	11	16	NA	80.7 ± 10.48	NA
IV paracetamol	35.19 ± 10.77	12	15	NA	79.19 ± 10.14	NA
Rajoria *et al*^[^19^]^						
IV ketorolac	38.50 ± 11.87	8	22	55.47 ± 5.07	83.67 ± 8.57	95.4 ± 7.65
IV paracetamol	41 ± 11.80	4	26	57.33 ± 6.95	82.53 ± 7.45	95.55 ± 6.67
Shaheen *et al*^[^20^]^						
IV ketorolac	36.20 ± 12.55	NA	NA	56.30 ± 9.44	NA	NA
IV paracetamol	34.40 ± 11.12	NA	NA	57.67 ± 8.13	NA	NA
Neupane *et al*^[^21^]^						
IV ketorolac	44.54 ± 12.16	13	22	66.54 ± 10.99	83.69 ± 14.37	94.94 ± 15.19
IV paracetamol	45.03 ± 10.81	7	28	61.60 ± 13.01	83.71 ± 16.71	95.57 ± 14.21
Ramakrishnan *et al*^[^22^]^						
IV ketorolac	38.5 ± 10.5	17	13	NA	NA	72.07 ± 6.8
IV paracetamol	39.47 ± 10.47	17	13	NA	NA	70.3 ± 7.83

SD, standard deviation; MAP, mean arterial pressure; IV, intravenous.

### Outcome

#### Primary outcome

The predefined primary outcome was the VAS score, assessed at multiple intervals to compare the analgesic efficacy of IV ketorolac versus IV paracetamol in postoperative pain relief.

Statistical analysis demonstrated that IV ketorolac was significantly associated with lower VAS scores at 2 hours (MD = −0.95, CI = −1.17 to −0.74, *P* < 0.00001), 4 hours (MD = −0.99, CI = −1.62 to −0.35, *P* = 0.002), and 8 hours (MD = −0.45, CI = −0.78 to −0.13, *P* = 0.006) postoperatively. Although lower VAS scores were also observed in the ketorolac group at 15 minutes (MD = −0.03, CI = −0.14 to 0.07, *P* = 0.53), 45 minutes (MD = −0.78, CI = −2.01 to 0.44, *P* = 0.21), 6 hours (MD = −0.08, CI = −0.66 to 0.50, *P* = 0.78), and 24 hours (MD = −0.32, CI = −0.90 to 0.25, *P* = 0.27), none reached statistical significance (all *P* > 0.05). These findings are summarized in Table 3.

**Table 3:** Primary outcomes evaluated in the meta-analysis: effect sizes, P values, and heterogeneity across outcomes.

Outcome (time/VAS) mean SD	No. of studies	Effect model	MD/OR	*I*^2^(%)	Ph	Effect size (95% CI)	*P* value
15 minutes	3	FE	MD	0	0.69	−0.03(−0.14, 0.07)	0.53
45 minutes	3	RE	MD	98	<0.00001	−0.78(−2.01, 0.44)	0.21
2 hours	4	FE	MD	0	0.46	−0.95(−1.17, −0.74)	**<0.00001**
4 hours	4	RE	MD	83	0.0004	−0.99(−1.62, −0.35)	**0.002**
6 hours	5	RE	MD	91	<0.00001	−0.08(−0.66, 0.50)	0.78
8 hours	2	FE	MD	0	0.71	−0.45(−0.78, −0.13)	**0.006**
24 hours	2	RE	MD	76	0.04	−0.32(−0.90, 0.25)	0.27

*P*-values <0.05 are highlighted in bold.

FE, fixed effect; RE, random effect; OR, odds ratio; MD, mean difference; Ph, *P* value of heterogeneity; VAS, visual analog scale.

##### VAS score at 15 minutes postoperatively

In RCTs (the only subgroup), the MD for VAS score at 15 minutes was −0.03 (95% CI: −0.14 to 0.07), indicating essentially no difference between IV paracetamol and IV ketorolac (*Z* = 0.63, *P* = 0.53). No heterogeneity was present (*I*^2^ = 0%) across the three RCTs, with Rastogi 2016 not estimable because a usable SD was not available at this timepoint. The overall/cumulative effect was identical to the RCT subgroup: MD −0.03 (95% CI: −0.14 to 0.07), *P* = 0.53. These data indicate that both drugs provide equivalent early pain control at 15 minutes postoperatively, likely reflecting that neither has reached peak analgesic effect at this time point (Fig. 2).

**Figure 2.**
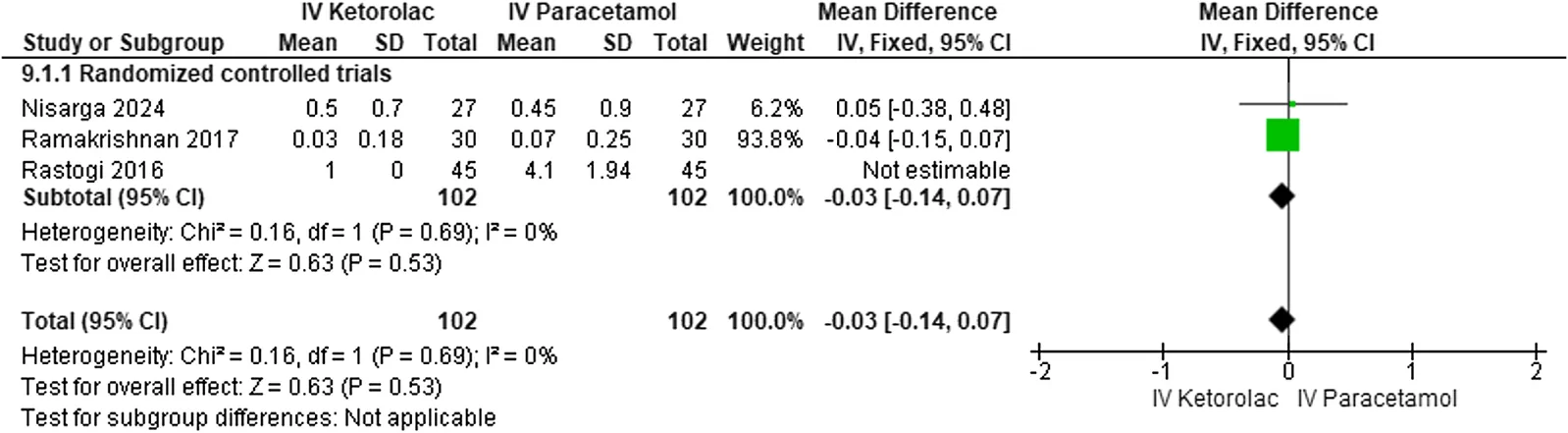
Forest plot for IV ketorolac versus IV paracetamol for VAS score at 15 minutes postoperatively, showing subgroup analysis according to RCTs. In this and all subsequent forest plots, IV ketorolac is the experimental arm and IV paracetamol the comparator; estimates to the left of the line of no effect favor IV ketorolac, except for time to first rescue analgesia, where estimates to the right favor IV ketorolac.

##### VAS score at 45 minutes postoperatively

In RCTs (the only subgroup), the MD was −0.78 (95% CI: −2.01 to 0.44), indicating a moderate trend toward lower VAS scores with IV ketorolac, but the CI broadly crossed zero and the effect was not significant (*Z* = 1.25, *P* = 0.21). Extreme heterogeneity was present (*I*^2^ = 98%), with Rastogi 2016 showing a large MD of −2.03, while Nisarga 2024 and Ramakrishnan 2017 showed near-zero differences. The overall (cumulative) effect was identical to the subgroup: MD = −0.78 (95% CI: −2.01 to 0.44), *P* = 0.21. These results indicate no statistically significant difference at 45 minutes, with extreme statistical inconsistency between studies, rendering this estimate unreliable (Fig. 3).

**Figure 3.**
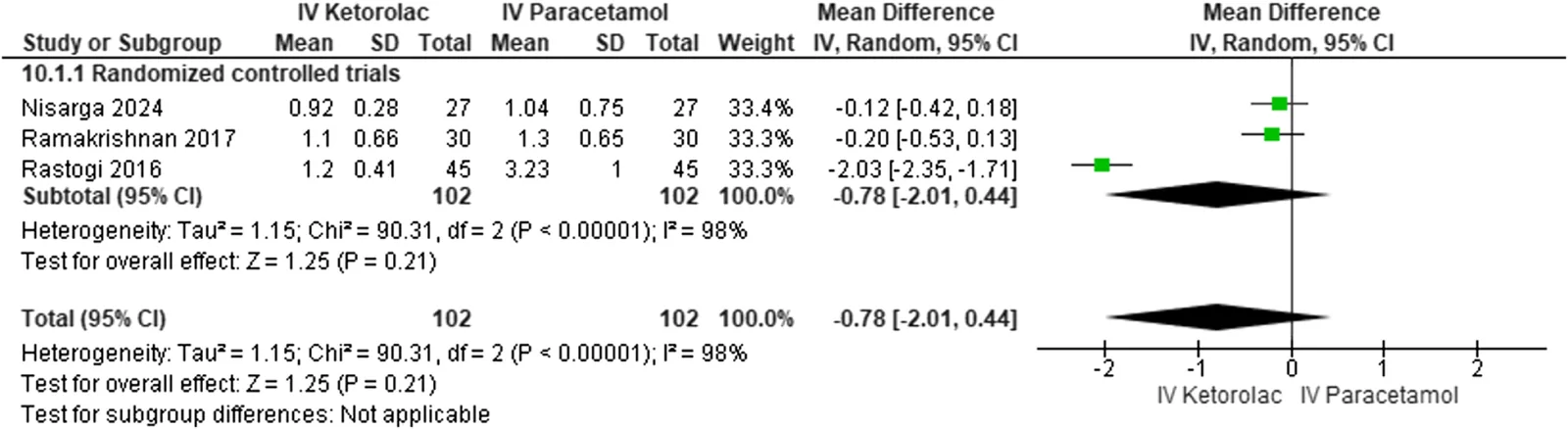
Forest plot of IV ketorolac versus IV paracetamol for the VAS score at 45 minutes postoperatively, showing subgroup analysis of RCTs.

##### VAS score at 2 hours postoperatively

In randomized controlled trials (the only subgroup), the MD was −0.95 (95% CI: −1.17 to −0.74), indicating a clinically meaningful and statistically robust reduction in pain scores with IV ketorolac at 2 hours (*Z* = 8.83, *P* < 0.00001). Across all four RCTs, no heterogeneity was present (*I*^2^ = 0%), indicating an exceptionally consistent signal with agreement in both direction and magnitude. The overall (cumulative) effect was identical to the RCT subgroup: MD −0.95 (95% CI: −1.17 to −0.74), *P* < 0.00001. These findings indicate that IV ketorolac significantly reduces VAS pain scores at 2 hours compared with IV paracetamol, representing the most consistent VAS result across the meta-analysis (Fig. 4).

**Figure 4.**
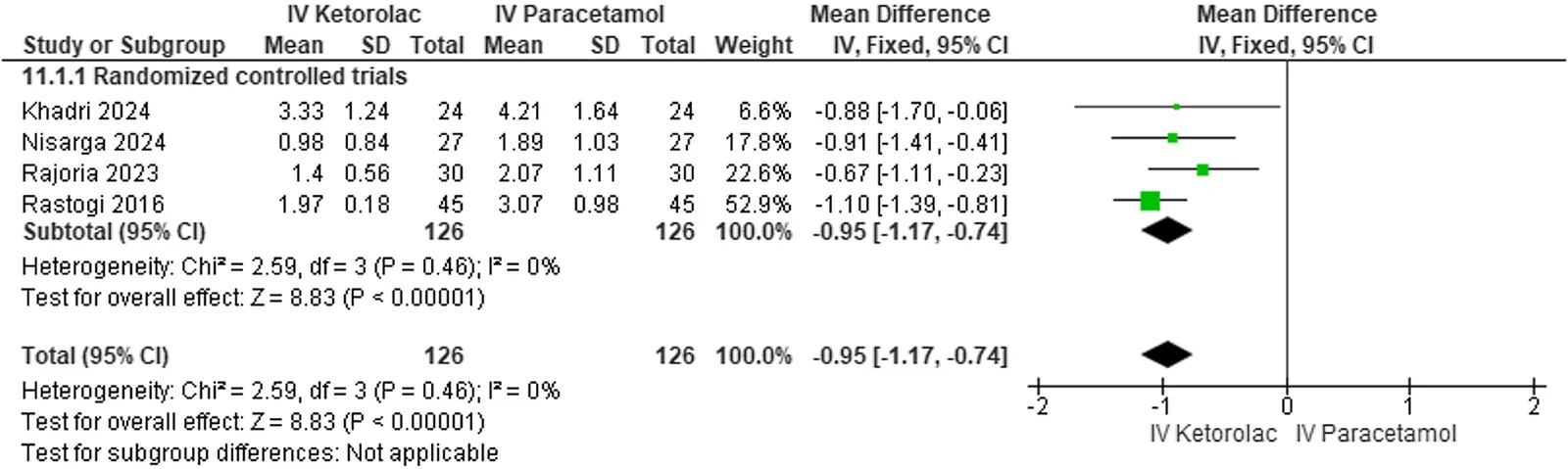
Forest plot of IV ketorolac versus IV paracetamol for VAS scores at 2 hours postoperatively, showing a subgroup analysis based on RCTs.

##### VAS score at 4 hours postoperatively

In RCTs (the only subgroup), the MD was −0.99 (95% CI: −1.62 to −0.35), indicating a statistically significant reduction in pain with IV ketorolac at 4 hours (*Z* = 3.03, *P* = 0.002). Moderate-to-high heterogeneity was present (*I*^2^ = 83%), driven by variation in effect magnitude: Khadri 2024 and Nisarga 2024 showed minimal differences, whereas Rajoria 2023 and Rastogi 2016 showed larger advantages for ketorolac. The overall (cumulative) effect was identical to the RCT subgroup: MD −0.99 (95% CI: −1.62 to −0.35), *P* = 0.002. These data indicate that IV ketorolac provides significantly lower VAS scores at 4 hours across RCTs, though the degree of heterogeneity signals that the magnitude of benefit varies meaningfully between individual trials (Fig. 5).

**Figure 5.**
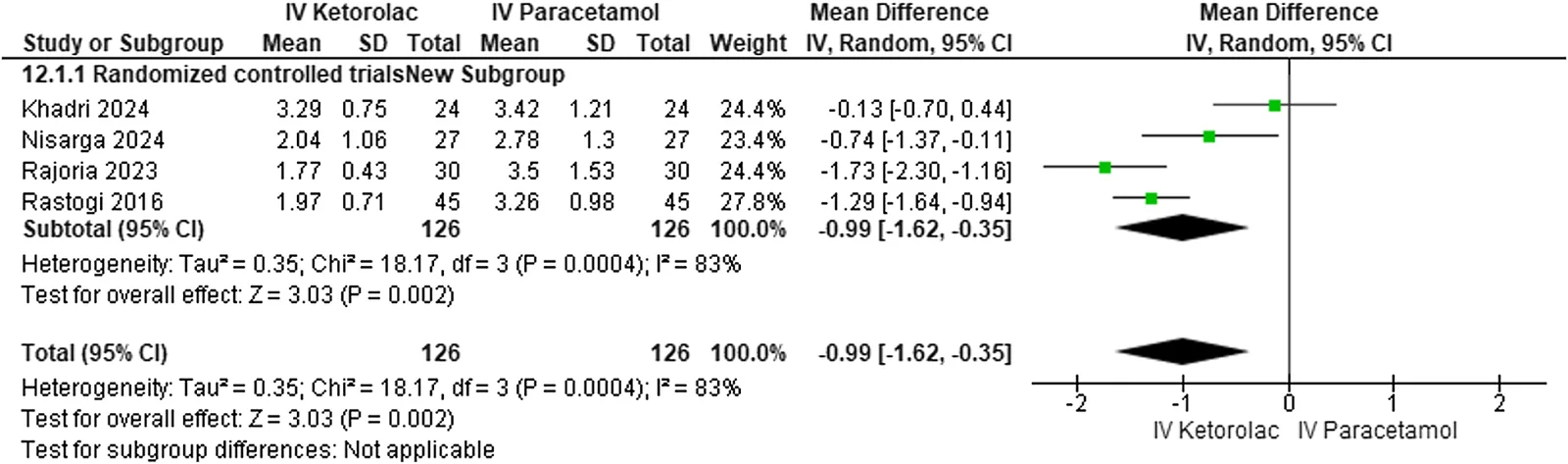
Forest plot of IV ketorolac versus IV paracetamol for the VAS score at 4 hours postoperatively, showing subgroup analysis across RCTs.

##### VAS score at 6 hours postoperatively

In RCTs (the only subgroup), the pooled MD for VAS score at 6 hours was −0.08 (95% CI: −0.66 to 0.50), indicating a numerically lower pain score with IV ketorolac that did not reach statistical significance (*Z* = 0.27, *P* = 0.78). Considerable heterogeneity was present (*I*^2^ = 91%; Chi^2^ = 43.79, df = 4, *P* < 0.00001), and a random-effects model was therefore applied. The inconsistency was driven almost entirely by Rajoria 2023, the only trial favoring IV paracetamol (MD +1.90; 95% CI: 1.20–2.60), whereas Khadri 2024, Nisarga 2024, Ramakrishnan 2017, and Rastogi 2016 all favored IV ketorolac. The overall/cumulative effect was identical to the RCT subgroup: MD −0.08 (95% CI: −0.66 to 0.50), *P* = 0.78. These data indicate that no statistically significant difference in VAS score at 6 hours can be demonstrated in the primary pooled analysis; the corresponding leave-one-out analysis excluding Rajoria 2023 is reported in Section 3.6 and Supplemental Digital Content Figure 13, available at: https://links.lww.com/MS9/B435 (Fig. 6).

**Figure 6.**
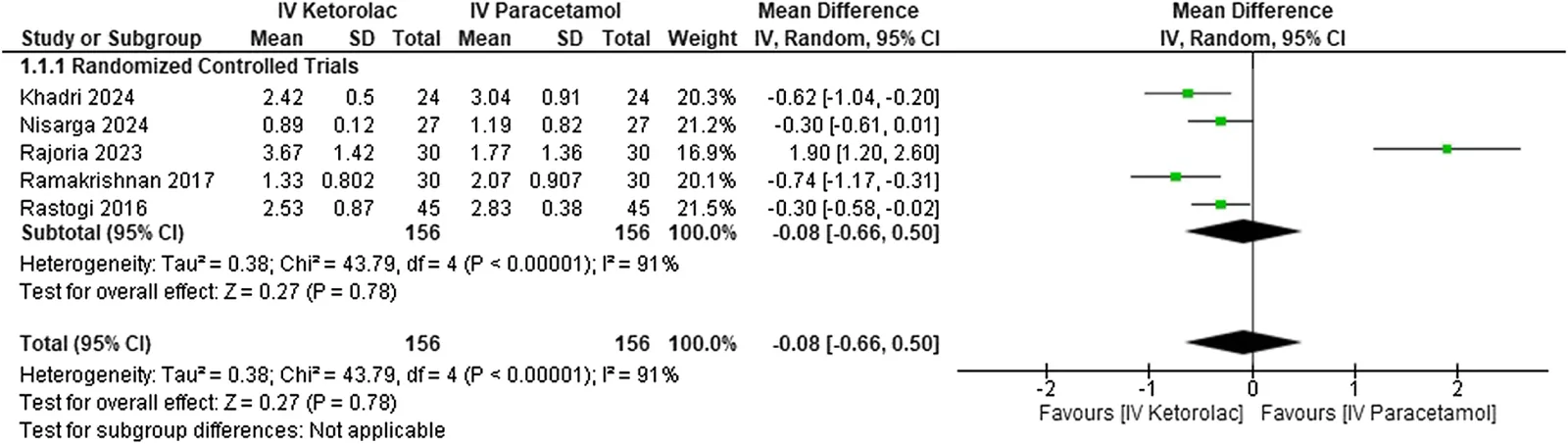
Forest plot of IV ketorolac versus IV paracetamol for VAS scores at 6 hours postoperatively, showing subgroup analyses based on RCTs.

##### VAS score at 8 hours postoperatively

In RCTs (the only subgroup), the MD was −0.45 (95% CI: −0.78 to −0.13), indicating that IV ketorolac significantly lowers pain scores at 8 hours (*Z* = 2.76; *P* = 0.006). No heterogeneity was present (*I*^2^ = 0%) between the two contributing RCTs (Khadri 2024, Nisarga 2024), both of which consistently favored ketorolac. The overall/cumulative effect was identical to the subgroup: MD = −0.45 (95% CI: −0.78 to −0.13), *P* = 0.006. These data indicate that IV ketorolac maintains a significant pain advantage at 8 hours, with consistent RCT evidence and no heterogeneity (Supplemental Digital Content Appendix 1, available at: https://links.lww.com/MS9/B435; Supplemental Digital Content Figure 1, available at: https://links.lww.com/MS9/B435).

##### VAS score at 24 hours postoperatively

In RCTs (the only subgroup), the MD for VAS score at 24 hours was −0.32 (95% CI: −0.90 to 0.25), indicating a trend toward lower pain scores with IV ketorolac compared to IV paracetamol, but the CI crossed zero and the effect was not statistically significant (*Z* = 1.11, *P* = 0.27). Substantial heterogeneity was present (*I*^2^ = 76%), with Nisarga 2024 showing a larger advantage for IV ketorolac (MD −0.66), whereas Rajoria 2023 showed almost no difference (MD −0.07). The overall (cumulative) effect was identical to the RCT subgroup: MD −0.32 (95% CI: −0.90 to 0.25), *P* = 0.27. These results indicate that the analgesic advantage of IV ketorolac does not persist as a statistically significant effect at 24 hours in RCTs; the effect attenuates over time, and the overall result is inconclusive due to high heterogeneity (Supplemental Digital Content Appendix 1, available at: https://links.lww.com/MS9/B435; Supplemental Digital Content Figure 2, available at: https://links.lww.com/MS9/B435).

#### Secondary outcome

The secondary outcomes evaluated were the time to first rescue analgesia; total consumption of rescue analgesics; number of patients requiring rescue analgesia; MAP and HR 60 minutes after intubation and HR 2 hours postoperatively; and the incidence of adverse effects such as nausea and vomiting.

The time to first rescue analgesia was significantly longer in the IV ketorolac group (MD = 1.93, CI = 1.26–2.61, *P* ≤ 0.00001). Fewer patients required rescue analgesia in this group (OR = 0.10, CI = 0.01–1.39, *P* = 0.09), and their total consumption of rescue analgesics was also lower (MD = −10.90, CI = −81.40–59.60, *P* = 0.76); however, neither of these two differences reached statistical significance, and both were affected by extreme between-study heterogeneity (*I*^2^ = 90 and 99%, respectively).

At 60 minutes after intubation, MAP was lower (MD = −1.02, CI = −3.09 to 1.04, *P* = 0.33) and HR was higher (MD = 1.65, CI = −2.40 to 5.70, *P* = 0.42) in the IV ketorolac group. Similarly, HR measured 2 hours postoperatively was higher (MD = 1.62, CI = −0.93 to 4.16, *P* = 0.21). However, none of these hemodynamic differences reached statistical significance (all *P* > 0.05).

Among adverse effects, the incidence of vomiting was significantly lower in the IV ketorolac group (OR = 0.05, CI = 0.01–0.42, *P* = 0.005). Nausea was also less frequent with IV ketorolac (OR = 0.36, CI = 0.09–1.39, *P* = 0.14), though this association was not statistically significant. No vomiting episodes were recorded in the ketorolac arms. These findings are detailed in Table 4.

**Table 4:** Secondary outcomes evaluated in the meta-analysis: effect sizes, P values, and heterogeneity across outcomes.

Outcome	No. of studies	Effect model	MD/OR	*I*^2^(%)	Ph	Effect size (95% CI)	*P* value
Time to first rescue analgesia (hours)	5	RE	MD	88	<0.00001	1.93 (1.26, 2.61)	**<0.00001**
Total consumption of rescue analgesics (mg)	2	RE	MD	99	<0.00001	−10.90 (−81.40, 59.60)	0.76
Total number of patients requiring rescue analgesics (*n*)	5	RE	OR	90	<0.00001	0.10 (0.01, 1.39)	0.09
MAP 60 minutes after intubation (mm of Hg)	3	FE	MD	0	0.57	−1.02 (−3.09, 1.04)	0.33
HR 60 minutes after intubation (bpm)	3	RE	MD	51	0.13	1.65 (−2.40, 5.70)	0.42
HR 2-hour post op (bpm)	2	FE	MD	0	0.90	1.62 (−0.93, 4.16)	0.21
Adverse effects: nausea (*n*)	3	FE	OR	29	0.24	0.36 (0.09, 1.39)	0.14
Adverse effects- vomiting (*n*)	3	FE	OR	0	0.96	0.05 (0.01, 0.42)	**0.005**

*P*-values <0.05 are highlighted in bold.

FE, fixed effect; RE, random effect; OR, odds ratio; MD, mean difference; Ph, *P* value of heterogeneity.

##### Time to first rescue analgesia

In RCTs, the MD = +1.98 hours (95% CI: 1.18–2.79), indicating that patients receiving IV ketorolac waited significantly longer before needing their first rescue analgesic (nearly 2 hours more; *Z* = 4.82, *P* < 0.00001). However, substantial heterogeneity was present (*I*^2^ = 91%), reflecting variation in the magnitude of benefit across the four RCTs (Khadri 2024, Nisarga 2024, Rajoria 2023, Rastogi 2016), all of which individually favored ketorolac. In the cohort study subgroup (Neupane 2024), the MD = +1.76 hours (95% CI: 0.98–2.54), indicating that patients also waited longer before needing rescue analgesia with IV ketorolac (*Z* = 4.42, *P* < 0.0001). The overall (cumulative) effect was MD = +1.93 hours (95% CI: 1.26–2.61), *Z* = 5.65, *P* < 0.00001, which was strongly significant, with high overall heterogeneity (*I*^2^ = 88%) but consistent directionality. The subgroup difference test was not significant (*P* = 0.70, *I*^2^ = 0%), indicating that RCTs and cohort studies agree on the direction of effect. These results indicate that IV ketorolac significantly prolongs time to first rescue analgesia by approximately 1.9 hours, a finding supported by both RCT and cohort evidence. (Supplemental Digital Content Appendix 1, available at: https://links.lww.com/MS9/B435, Supplemental Digital Content Figure 3, available at: https://links.lww.com/MS9/B435).

##### Total rescue analgesic consumption

In RCTs (single trial, Nisarga 2024), the MD for total rescue analgesic consumption was −46.94 mg (95% CI: −55.26 to −38.62), indicating that IV paracetamol patients consumed significantly more rescue analgesic than IV ketorolac patients (a large effect favoring ketorolac; Z = 11.05, *P* < 0.00001). In the cohort study subgroup (single trial, Neupane 2024), the MD was +25.00 mg (95% CI: 19.43–30.57), indicating the opposite pattern: IV ketorolac patients consumed 25 mg more rescue analgesic than IV paracetamol patients, favoring paracetamol (*Z* = 8.80, *P* < 0.00001). The overall/cumulative effect was MD −10.90 mg (95% CI: −81.40 to 59.60), *Z* = 0.30, *P* = 0.76, completely non-significant, with extreme heterogeneity (*I*^2^ = 99%) and a highly significant subgroup difference test (Chi^2^ = 198.27, *P* < 0.00001, *I*^2^ = 99.5%). These data indicate that the RCT and cohort results are irreconcilably contradictory, rendering the pooled outcome uninterpretable and highlighting that study design is a critical effect modifier for total rescue analgesic consumption (Supplemental Digital Content Appendix 1, available at: https://links.lww.com/MS9/B435, Supplemental Digital Content Figure 4, available at: https://links.lww.com/MS9/B435).

##### Total number of patients requiring rescue analgesia

In RCTs, the pooled OR was 0.04 (95% CI: 0.00–1.08), indicating a dramatic point estimate favoring IV ketorolac (far fewer patients needed rescue analgesia). However, the wide CI crossed 1.0, and the effect was borderline non-significant (*Z* = 1.91, *P* = 0.06). Very high heterogeneity was present (*I*^2^ = 87%), with Shaheen 2017 reporting an OR of 1.38 (favoring paracetamol) and the remaining RCTs strongly favoring ketorolac; a random-effects model was applied. In the cohort study subgroup (only one study, Neupane 2024), the OR was 1.59 (95% CI: 0.62–4.09), indicating a non-significant trend toward more patients needing rescue analgesia in the IV ketorolac group (*Z* = 0.96, *P* = 0.34). The overall (cumulative) effect was OR 0.10 (95% CI: 0.01–1.39), *Z* = 1.71, *P* = 0.09, also non-significant, with extreme heterogeneity (*I*^2^ = 90%) and a significant subgroup difference test (Chi^2^ = 4.44, *P* = 0.04, *I*^2^ = 77.5%). These findings indicate no statistically significant overall difference in the proportion of patients requiring rescue analgesia, with RCTs tending to favor IV ketorolac and the single cohort study tending to favor IV paracetamol, precluding a reliable pooled interpretation (Supplemental Digital Content Appendix 1, available at: https://links.lww.com/MS9/B435, Supplemental Digital Content Figure 5, available at: https://links.lww.com/MS9/B435).

##### MAP at 60 minutes post-intubation

In RCTs, the MD for MAP was −1.30 mmHg (95% CI: −3.48 to 0.88), indicating a small trend toward lower MAP with IV ketorolac, but the CI crossed zero and the effect was not significant (*Z* = 1.17, *P* = 0.24). No heterogeneity was present (*I*^2^ = 0%) across the two RCTs. In the cohort study subgroup (Neupane 2024), the MD was +1.30 mmHg (95% CI: −5.03 to 7.63), indicating a non-significant trend toward higher MAP with IV ketorolac (*Z* = 0.40, *P* = 0.69). The overall/cumulative effect was MD −1.02 mmHg (95% CI: −3.09 to 1.04), Z = 0.97, *P* = 0.33, with no overall heterogeneity (*I*^2^ = 0%). The subgroup difference test was not significant (*P* = 0.45), indicating that both study designs were compatible. These findings indicate no statistically significant difference in MAP at 60 minutes post-intubation, with hemodynamic equivalence supported consistently across RCTs and cohort studies (Supplemental Digital Content Appendix 1, available at: https://links.lww.com/MS9/B435, Supplemental Digital Content Figure 6, available at: https://links.lww.com/MS9/B435).

##### Heart rate at 60 minutes post-intubation

In RCTs, the MD was +0.86 beats/minutes (95% CI: −5.65 to 7.37), indicating a non-significant trend toward higher HR with IV ketorolac (*Z* = 0.26, *P* = 0.80). High heterogeneity was present (*I*^2^ = 75%), with Nisarga 2024 showing lower HR with ketorolac (MD = −2.85) and Rastogi 2016 showing higher HR with ketorolac (MD = +3.83); a random-effects model was applied. In the cohort study subgroup (Neupane 2024), the MD was +2.96 beats/minutes (95% CI: −3.02 to 8.94), indicating a non-significant trend toward higher HR with IV ketorolac (*Z* = 0.97, *P* = 0.33). The overall/cumulative effect was MD = +1.65 (95% CI: −2.40 to 5.70), *Z* = 0.80, *P* = 0.42, with moderate overall heterogeneity (*I*^2^ = 51%). The subgroup difference test was not significant (*P* = 0.64, *I*^2^ = 0%), indicating that RCTs and cohort studies were directionally compatible. These results indicate no statistically significant difference in HR at 60 minutes post-intubation by either study design, though within-RCT heterogeneity suggests cautious interpretation (Supplemental Digital Content Appendix 1, available at: https://links.lww.com/MS9/B435, Supplemental Digital Content Figure 7, available at: https://links.lww.com/MS9/B435).

##### Heart rate at 2 hours postoperatively

In RCTs (the only study-design subgroup), the MD in HR at 2 hours was +1.62 beats/minutes (95% CI: −0.93 to 4.16) for IV ketorolac versus IV paracetamol, indicating a slightly higher HR with ketorolac that was not statistically significant (*Z* = 1.25, *P* = 0.21). No heterogeneity was present (*I*^2^ = 0%) across the two RCTs (Nisarga 2024, Rajoria 2023), indicating consistent agreement on a null result. The overall/cumulative effect was identical to the RCT subgroup: MD +1.62 (95% CI: −0.93 to 4.16), *P* = 0.21. These data indicate that RCTs find no significant difference in HR at 2 hours postoperatively between IV ketorolac and IV paracetamol, with a clinically trivial effect size (Supplemental Digital Content Appendix 1, available at: https://links.lww.com/MS9/B435, Supplemental Digital Content Figure 8, available at: https://links.lww.com/MS9/B435).

##### Postoperative nausea

In RCTs (the only subgroup), the pooled OR for postoperative nausea was 0.36 (95% CI: 0.09–1.39), indicating a trend toward lower odds of nausea with IV ketorolac; however, the CI crossed 1.0, and the effect was not statistically significant (*Z* = 1.48, *P* = 0.14). Moderate heterogeneity was present (*I*^2^ = 29%), partly driven by Ramakrishnan 2017 (OR >1, favoring paracetamol), while the other two RCTs favored ketorolac. The overall (cumulative) effect was the same as the RCT subgroup: OR 0.36 (95% CI: 0.09–1.39), *P* = 0.14. These results indicate no statistically significant difference between IV ketorolac and IV paracetamol for postoperative nausea in RCTs, with only a non-significant trend favoring ketorolac (Supplemental Digital Content Appendix 1, available at: https://links.lww.com/MS9/B435, Supplemental Digital Content Figure 9, available at: https://links.lww.com/MS9/B435).

##### Postoperative vomiting

In RCTs (the only study-design subgroup), the pooled OR for postoperative vomiting was 0.05 (95% CI: 0.01–0.42) for IV ketorolac versus IV paracetamol, indicating substantially lower odds of vomiting with IV ketorolac than with IV paracetamol. The CI did not cross 1.0, and the effect was statistically significant (*Z* = 2.79, *P* = 0.005). Heterogeneity was negligible (*I*^2^ = 0%); however, only two of the three RCTs contributed data, as Ramakrishnan 2017 was not estimable because no vomiting occurred in either arm. The overall (cumulative) effect was identical to the RCT-only estimate: OR = 0.05 (95% CI: 0.01–0.42), *P* = 0.005. These data indicate that the available RCT evidence favors IV ketorolac with respect to postoperative vomiting; because the estimate rests on zero events in the ketorolac arms and a total of only 14 events overall, it should be read as indicating the direction of effect rather than a reliable estimate of its magnitude (Supplemental Digital Content Appendix 1, available at: https://links.lww.com/MS9/B435, Supplemental Digital Content Figure 10, available at: https://links.lww.com/MS9/B435).

### Risk of bias in individual studies

Risk of bias was evaluated using the RoB 2 tool for RCTs. Four studies demonstrated a low risk of bias across key domains, while three were judged to have some concerns. The single cross-sectional study was assessed using the Newcastle-Ottawa Scale and was rated as high quality, receiving 8 stars. Overall, the evidence from these studies can be considered reliable for drawing conclusions, as illustrated in the supplementary files (Supplemental Digital Content Appendix 1, available at: https://links.lww.com/MS9/B435, and Supplemental Digital Content Figures 11 and 12, available at: https://links.lww.com/MS9/B435).

### Publication bias

Given that Egger’s test^[^23^]^ has limited statistical power when applied to fewer than ten studies, current methodological guidelines advise against its use in such cases. As our analysis included only eight studies, we did not perform Egger’s test^[^23^]^ to assess publication bias. Funnel-plot asymmetry could not be evaluated reliably for the same reason; small-study effects and publication bias therefore cannot be excluded and are acknowledged among the limitations of this review.

#### Assessment of heterogeneity with respect to type of surgeries

Across the 15 outcomes examined, the relationship between the individual surgery-type subgroup estimates and the cumulative pooled effect falls into the following categories:

Concordant: postoperative vomiting; postoperative nausea; HR at 2 hours; MAP at 60 minutes; VAS at 15 minutes, 2 hours, and 8 hours. For these outcomes, only a single surgical subgroup contributed data or both subgroups yielded consistent results, so the cumulative estimate faithfully represents the underlying data.

Concordant with notable magnitude difference: time to first rescue analgesia and VAS at 6 hours. For time to first rescue analgesia, both subgroups agree on direction and significance. For VAS at 6 hours, both subgroups favor IV ketorolac in direction, but only the “any laparoscopic surgery” subgroup reaches significance (*P* = 0.006), while the cholecystectomy subgroup does not (*P* = 0.68). In both outcomes, the surgery-type analysis reveals that the magnitude of benefit differs between cholecystectomy and any LS, information the cumulative estimate alone does not convey.

Cumulative masks a subgroup-level pattern: HR at 60 minutes post-intubation; VAS at 4 and 24 hours; and the total number of patients requiring rescue analgesia. In these outcomes, the cumulative result is either technically correct yet misleading (e.g., a significant overall result driven entirely by one subgroup), or a non-significant overall result that conceals a significant or divergent subgroup finding.

Opposite directions: total rescue analgesic consumption. The two contributing studies represent opposite surgery types and report opposite effects of equal magnitude, rendering the pooled estimate both statistically and clinically meaningless.

The most clinically critical finding is for HR at 60 minutes post-intubation: the laparoscopic cholecystectomy subgroup shows a statistically significant increase in HR with IV ketorolac (MD, +3.61 bpm; 95% CI: 0.63 to 6.60; *P* = 0.02) that is entirely nullified in the cumulative pool by the opposing trend from the “any laparoscopic surgery” subgroup. This masked hemodynamic signal would be missed entirely if only the cumulative result were reported. For VAS at 4 hours and VAS at 24 hours, the subgroup analysis reveals that the overall significant or non-significant conclusion depends heavily on surgery type: at 4 hours, the benefit is concentrated in cholecystectomy; at 24 hours, a significant benefit in general LS disappears specifically in cholecystectomy. Both findings carry implications for how the results should be communicated to clinicians treating patients undergoing different types of laparoscopic procedures. The complete analysis is provided in the supplementary file (Supplemental Digital Content Appendix 2, available at: https://links.lww.com/MS9/B436, Supplemental Digital Content Figures 1–15, available at: https://links.lww.com/MS9/B436).

### Sensitivity analysis

Sensitivity analysis revealed substantial heterogeneity across several variables, with VAS scores at 45 minutes, 4 hours, 6 hours, and 24 hours demonstrating significant heterogeneity (*I*^2^ > 75%). Among the secondary outcomes, considerable heterogeneity was also observed in the time to first rescue analgesia, the total consumption of rescue analgesics, and the number of patients requiring rescue analgesia.

These outcomes were further evaluated using a leave-one-out analysis, systematically excluding individual studies initially included to assess their impact, as shown in Table 5. Notably, after excluding the Rajoria *et al*^[^19^]^ study, the difference in VAS scores at 6 hours postoperatively, which was initially non-significant, became statistically significant, with a lower score in the IV ketorolac group (*P* < 0.05), as shown in Supplemental Digital Content Appendix 1, available at: https://links.lww.com/MS9/B435 and Supplemental Digital Content Figure 13, available at: https://links.lww.com/MS9/B435.

**Table 5:** Leave-one-out analysis.

Outcome	No. of studies	Initial *I*^2^ (%)	Initial Ph	Initial *P* value	Study removed	Final *I*^2^ (%)	Final Ph	Final *P* value
Time/VAS mean SD								
45 minutes	3	98	<0.00001	0.21	Rastogi 2016^[^15^]^	0	0.73	0.17
4 hours	4	83	0.0004	0.002	Khadri 2024^[^16^]^	62	0.07	<0.00001
6 hours	5	91	<0.00001	0.78	Rajoria 2023^[^19^]^	30	0.23	<0.00001
24 hours^a^	2	76	0.04	0.27	–	–	–	–
Time to first rescue analgesia (h)	5	88	<0.00001	<0.00001	Rastogi 2016^[^15^]^	0	0.43	<0.00001
Total consumption of rescue analgesics (mg)^a^	2	99	<0.00001	0.76	–	–	–	–
Total number of patients requiring rescue analgesic (*n*)	5	90	<0.00001	0.09	Rastogi 2016^[^15^]^	86	0.0001	0.24

^a^ Since there are only two studies, a leave-one-out analysis isn’t feasible

## Discussion

This systematic review and meta-analysis evaluated and compared intraoperative and postoperative outcomes associated with IV ketorolac and IV paracetamol in patients undergoing LSs. The VAS scores at 2, 4, and 8 hours postoperatively demonstrated statistically significant differences between the groups, with lower scores observed in the IV ketorolac group. Conversely, VAS scores at 15 minutes, 45 minutes, 6 hours, and 24 hours were not significantly different between the two groups. However, after exclusion of the study by Rajoria *et al*., the difference in VAS scores at 6 hours, previously non-significant, became statistically significant between the groups, with lower scores observed in the IV ketorolac group. Among the secondary outcomes, the time to first rescue analgesia was significantly longer with IV ketorolac (MD = 1.93 hours; 95% CI: 1.26–2.61; *P* < 0.00001), and postoperative vomiting was significantly less frequent (OR = 0.05; 95% CI: 0.01–0.42; *P* = 0.005). In contrast, total rescue analgesic consumption, the number of patients requiring rescue analgesia, MAP and HR at 60 minutes post-intubation, HR at 2 hours postoperatively, and the incidence of nausea did not differ significantly between the two drugs. Additionally, subgroup analyses based on the type of LS and study design demonstrated that certain outcomes varied according to procedural context. While some findings remained consistent across subgroups, outcomes such as VAS scores at 4 and 24 hours, HR at 60 minutes post-intubation, and rescue analgesia requirements exhibited differences in effect magnitude or direction between laparoscopic cholecystectomy and mixed laparoscopic procedures. These observations suggest that the relative analgesic benefits of IV ketorolac and IV paracetamol may partly depend on the surgical type and the underlying study characteristics.

The findings of this meta-analysis are consistent with and supported by previous studies in the literature. Several studies have shown that IV ketorolac is associated with improved early postoperative analgesia compared with IV paracetamol. Zohrevandi *et al*^[^24^]^ reported significantly lower VAS scores in the ketorolac group during the early postoperative phase, a finding corroborated by Shakeri *et al*^[^25^]^. The enhanced analgesic effect of ketorolac can be attributed to its pharmacological mechanism as an NSAID. Ketorolac inhibits cyclooxygenase (COX) enzymes, leading to reduced synthesis of prostaglandins (PGs), which are key mediators of inflammation and nociception. Consequently, ketorolac diminishes both peripheral and central sensitization, thereby producing a more potent analgesic response and reducing postoperative pain intensity^[^26,27^]^.

Conversely, paracetamol primarily exerts its analgesic action via central mechanisms. It inhibits COX-2 activity within the brain, modulates serotonergic descending pain pathways, and may also impede N-methyl-D-aspartate receptor-mediated nitric oxide formation. However, its anti-inflammatory potential is limited because it does not substantially suppress peripheral PG synthesis. Thus, while paracetamol remains a safe and effective analgesic, its efficacy in managing inflammation-related pain is relatively modest compared to ketorolac, particularly during the immediate postoperative period^[^28,29^]^. Consistent with this, earlier trials have reported no significant difference between the two agents in the very early^[^30^]^ or the later postoperative phase^[^31^]^, mirroring the non-significant estimates we obtained at 15 and 45 minutes and at 24 hours. This temporal variation likely reflects pharmacokinetic disparities between the drugs: ketorolac achieves peak plasma concentrations within 30–60 minutes, whereas paracetamol demonstrates a slightly delayed but more sustained analgesic profile^[^32^]^. The subgroup analyses further support this temporal variability, as certain procedure-specific subgroups demonstrated differences in analgesic response at intermediate postoperative intervals, suggesting that surgical stimulus and operative complexity may influence the duration and magnitude of analgesic efficacy.

Total rescue analgesic consumption and the proportion of patients requiring additional analgesia did not differ significantly between the treatment groups, although both pooled estimates were affected by extreme heterogeneity (*I*^2^ = 99 and 90%, respectively) and are therefore uninformative rather than genuinely null. By contrast, the time to first rescue analgesia was significantly longer in the ketorolac group, by approximately 1.9 hours^[^33^]^. This pattern suggests that the advantage of ketorolac is concentrated in the early postoperative period, after which both drugs maintain broadly similar efficacy in preventing breakthrough pain^[^34^]^. Furthermore, hemodynamic parameters, including MAP and HR, did not significantly differ between the groups^[^35^]^, likely because both drugs exert minimal direct cardiovascular influence when administered within therapeutic ranges. These findings underscore their suitability as components of opioid-sparing protocols^[^36^]^. With the exception of postoperative vomiting, adverse effects were comparable between the two interventions^[^37^]^. Vomiting was significantly less frequent in the ketorolac group (OR = 0.05; *P* = 0.005), while nausea showed a non-significant trend in the same direction. The differential pattern in these outcomes may be explained by their pharmacodynamic profiles: while ketorolac acts peripherally via COX inhibition, paracetamol’s central action has limited impact on gastrointestinal or central mechanisms that trigger nausea and vomiting^[^38^]^. Nevertheless, subgroup evaluation revealed that certain hemodynamic findings, particularly HR at 60 minutes post-intubation, differed between laparoscopic cholecystectomy and broader LS cohorts, indicating that pooled overall estimates may occasionally mask subgroup-specific trends.

The results of this meta-analysis emphasize the utility of both IV ketorolac and IV paracetamol as non-opioid analgesics in LS. IV ketorolac was associated with better early postoperative pain control and a longer time to rescue analgesia, consistent with findings from double-blind trials showing pre-emptive ketorolac’s analgesic benefit over placebo in diverse surgical settings^[^39^]^. Conversely, IV paracetamol offers comparable overall analgesia with a more favorable safety profile, making it preferable for patients with renal, gastrointestinal, or bleeding risks^[^40^]^. Both agents are consistent with ERAS recommendations advocating the routine inclusion of non-opioid analgesics to minimize opioid consumption, expedite recovery, and improve postoperative comfort^[^41^]^. The selection between these drugs should, therefore, be individualized according to patient comorbidities, surgical complexity, and institutional practice, ensuring effective and safe postoperative pain control. Importantly, the subgroup analyses reinforce the need for individualized analgesic strategies, as the relative advantages of each agent may vary according to surgical subtype, perioperative pain burden, and patient-specific risk factors.

Despite these findings, several methodological limitations should be considered. The included studies exhibited heterogeneity in study design, type of surgical procedures, and timing and dosing protocols of analgesic administration, which may have introduced variability in outcomes and limited direct comparability across trials. To address this, we used subgroup analysis by study design to isolate the RCT data, which functioned as a sensitivity analysis to preserve the integrity of the randomized findings. Additionally, a systematic leave-one-out analysis was performed to transparently identify the specific studies driving statistical heterogeneity, thereby justifying the inclusion of diverse study designs without compromising the overall conclusions. Some outcomes were reported in a limited number of studies, further limiting the robustness of pooled analyses. The relatively small sample size may have reduced statistical power to detect subtle differences in analgesic efficacy. Because fewer than ten studies contributed to each outcome, formal statistical assessment of small-study effects and publication bias was not possible, so selective reporting cannot be excluded. Several outcomes, notably total rescue analgesic consumption and the number of patients requiring rescue analgesia, showed heterogeneity of such a degree (*I*^2^ = 99% and 90%) that the pooled estimates are best regarded as descriptive rather than valid summary effects. Consequently, the findings from our subgroup analyses should be interpreted with caution, as the limited number of contributing studies in each stratum reduces statistical power and limits the broad generalizability of these specific comparisons.

In addition, the short duration of follow-up limits evaluation of long-term safety outcomes. Pain assessment is inherently subjective and may be influenced by individual pain thresholds, psychological factors, and anesthetic depth, introducing potential bias. Biochemical markers of hepatic and renal function were not consistently assessed, limiting the comprehensiveness of the safety evaluation. Although no major adverse events were reported, the lack of systematic reporting of NSAID-related risks, including renal, gastrointestinal, and hemostatic effects, warrants cautious interpretation. Importantly, the absence of reported adverse events does not confirm the absence of harm, and future trials should adopt standardized safety reporting frameworks.

Furthermore, several of the included primary studies were single-center, which may limit external validity as findings may not be generalizable across different populations or surgical settings. The restriction to a specific surgical cohort limits applicability to other procedures, and variability in surgical techniques, duration of procedures, and intraoperative handling across studies may have further contributed to outcome heterogeneity. Procedural variability, including differences in intubation technique and operator experience, was not standardized and may have influenced postoperative discomfort.

Although the timing of analgesic administration was intended to be standardized, minor inter-study variations in administration timing and perioperative protocols may have influenced the onset and magnitude of analgesic effects. Hemodynamic assessment was limited to non-invasive monitoring, potentially overlooking subtle cardiovascular changes detectable with invasive methods. Additionally, patient-centered outcomes such as satisfaction, recovery quality, and overall comfort were not systematically evaluated. Potential confounders, including concurrent medications, anxiety levels, and prior analgesic exposure, were not controlled. Finally, the absence of an economic analysis limits understanding of cost-effectiveness, particularly in resource-limited clinical settings.

These findings contribute to the evolving evidence base supporting the use of non-opioid analgesics in minimally invasive surgery. However, to strengthen clinical guidelines and optimize postoperative pain management, large-scale, multicenter RCTs are required. Future research should standardize dosing regimens, timing of administration, and outcome assessments to minimize heterogeneity across studies. Additionally, trials should evaluate long-term safety outcomes, including renal and hepatic effects, and explore pharmacoeconomic aspects to determine cost-effectiveness in different healthcare settings. Integration of patient-centered outcomes, such as satisfaction, recovery quality, and functional recovery, will further clarify the clinical value of IV ketorolac and IV paracetamol within ERAS protocols.

Substantial statistical heterogeneity observed across primary outcomes in this meta-analysis can be attributed to several distinct clinical and methodological variations among the included studies. First, differences in surgical procedures and patient populations introduce inherent variability in pain pathophysiology, while the majority of trials evaluate laparoscopic cholecystectomy (Rastogi *et al*., Ramakrishnan *et al*., Rajoria *et al*., Neupane *et al*., Medina-Vera *et al*.), others include day-case gynecological laparoscopies (Shaheen *et al*.) or mixed laparoscopic abdominal procedures (Nisarga *et al*., Khadri *et al*.), which vary significantly in their somatic versus visceral pain burden and residual irritation. Second, timing of drug administration varies markedly: five studies evaluate true preemptive administration 30–60 minutes prior to surgical induction or incision, whereas others administer interventions post-induction (Shaheen *et al*.) or 30 minutes prior to surgical completion (Ramakrishnan *et al*.). Third, perioperative protocols and co-interventions create significant confounding; notably, Medina-Vera *et al*. routinely use pre-incisional bupivacaine infiltration at trocar sites and intraoperative remifentanil infusions, while Neupane *et al*. institute routine scheduled postoperative dosing of both study drugs (IV paracetamol 6-hourly and IV ketorolac 8-hourly) rather than relying solely on single-dose preemptive kinetics. Finally, study designs diverge in rescue analgesic regimens (ranging from IV tramadol to IV morphine or IM pethidine), threshold definitions for rescue intervention, and postoperative evaluation timelines (6 hours vs. 12 or 24 hours). These clinical, pharmacological, and procedural disparities explain the observed heterogeneity and underscore the impact of trial methodology on comparative analgesic outcomes.

## Conclusion

IV ketorolac appears to offer superior early postoperative analgesia compared with IV paracetamol and is additionally associated with a significantly longer time to first rescue analgesia and a lower incidence of postoperative vomiting, while both agents demonstrate comparable short-term safety and hemodynamic stability. However, given the short follow-up periods and inconsistent reporting of adverse events in the included trials, definitive conclusions regarding their broader safety profiles require further investigation. The findings suggest that ketorolac may be preferable when rapid and potent pain relief is needed, whereas paracetamol remains a valuable alternative for patients with NSAID contraindications. Both agents can be effectively incorporated into multimodal, opioid-sparing analgesic strategies for LS. However, the available evidence also highlights the importance of individualized analgesic selection based on patient comorbidities and surgical context, which may ultimately contribute to improved recovery and adherence to ERAS principles. Further well-designed RCTs are warranted to confirm these observations across a broader range of laparoscopic procedures and patient populations.

## Data Availability

All data analyzed in this study were obtained from previously published articles. The datasets generated and/or analyzed during the current study are available from the corresponding author upon reasonable request. All included studies are cited in the reference list.
